# Schizophrenia may be considered as a member of the spectrum of PBAFopathies

**DOI:** 10.1192/j.eurpsy.2023.761

**Published:** 2023-07-19

**Authors:** B. Abdelmoula, S. Sellami, W. Smaoui, N. Bouayed Abdelmoula

**Affiliations:** Genomics of Signalopathies at the service of Medicine, Medical University of Sfax, Sfax, Tunisia

## Abstract

**Introduction:**

Chromatin modifications and epigenetics are important pathogenesis mechanisms leading to various neurologic and psychiatric disorders including epilepsy, drug addictions, depression, autistic spectrum, learning disabilities and schizophrenia. Recently, the disruption of the chromatin remodeling BAF complex has been linked to several neurodevelopmental syndromes, commonly referred to as PBAFopathies.

**Objectives:**

Here, we review the implication of PBAF complex genes in schizophrenia and we outline syndromes caused by mutations in these chromatin-modifying enzymes labelled as PBAFopathies to discuss the functional consequences of reported mutations in the literature.

**Methods:**

We comprehensively review the scientific literature using Pubmed database and other search platforms such as Google scholar to state the role of PBAF complex genes in schizophrenia and to reveal the most frequent genes mutations reported in literature.

**Results:**

Our review revealed that the human analogs of the subfamily of ATP-dependent chromatin remodeling complexes, which are known in eukaryotes as the mammalian SWI/SNF complex (counting a group of proteins that associate and possess a DNA-stimulated ATPase activity that can destabilize histone-DNA interactions in reconstituted nucleosomes providing crucial nucleosome rearrangement and allowing the activation/repression of genes) are crucial for the regulation of genes expression and cells differentiation. They involve two well-known complexes which are SWI/SNF-A (known as BAF complex) and SWI/SNF-B (known as Polybromo-associated BAF or PBAF complex). SWI/SNF is a multisubunit chromatin-remodeling complex that performs fundamental roles in gene regulation, cell lineage specification, and organismal development and mutations that inactivate SWI/SNF subunits are found in nearly 20% of human cancers and in various developmental disorders, forming a continuum or spectrum of diseases. Since the first description of BRG1/BRM mutations in schizophrenia, other mutations of the SWI/SNF subunits have been reported: SMARCA1, SMARCA2, SMARCA4/BRG1, etc. Single nucleotide polymorphisms (SNPs) in these and other genes of PBAF have been also associated with schizophrenia.

**Conclusions:**

This review focuses on the PBAF SWI/SNF subunits to find out if schizophrenia may be considered as a member of the spectrum of PBAFopathies.

**Disclosure of Interest:**

None Declared

